# Continental scale patterns and predictors of fern richness and phylogenetic diversity

**DOI:** 10.3389/fgene.2015.00132

**Published:** 2015-04-14

**Authors:** Nathalie S. Nagalingum, Nunzio Knerr, Shawn W. Laffan, Carlos E. González-Orozco, Andrew H. Thornhill, Joseph T. Miller, Brent D. Mishler

**Affiliations:** ^1^National Herbarium of New South Wales, Royal Botanic Gardens and Domain TrustSydney, NSW, Australia; ^2^Centre for Australian National Biodiversity Research, Commonwealth Scientific and Industrial Research Organisation Plant IndustryCanberra, ACT, Australia; ^3^Centre for Ecosystem Science, School of Biological, Earth and Environmental Sciences, University of New South WalesSydney, NSW, Australia; ^4^Institute for Applied Ecology and Collaborative Research Network for Murray-Darling Basin Futures, University of CanberraACT, Australia; ^5^Australian Tropical Herbarium, James Cook UniversityCairns, QLD, Australia; ^6^University and Jepson Herbaria, and Department of Integrative Biology, University of CaliforniaBerkeley, CA, USA

**Keywords:** Polypodiopsida, Filicopsida, ferns, Australia, conservation, evolution, community, spatial phylogenetics

## Abstract

Because ferns have a wide range of habitat preferences and are widely distributed, they are an ideal group for understanding how diversity is distributed. Here we examine fern diversity on a broad-scale using standard and corrected richness measures as well as phylogenetic indices; in addition we determine the environmental predictors of each diversity metric. Using the combined records of Australian herbaria, a dataset of over 60,000 records was obtained for 89 genera to infer richness. A molecular phylogeny of all the genera was constructed and combined with the herbarium records to obtain phylogenetic diversity patterns. A hotspot of both taxic and phylogenetic diversity occurs in the Wet Tropics of northeastern Australia. Although considerable diversity is distributed along the eastern coast, some important regions of diversity are identified only after sample-standardization of richness and through the phylogenetic metric. Of all of the metrics, annual precipitation was identified as the most explanatory variable, in part, in agreement with global and regional fern studies. However, precipitation was combined with a different variable for each different metric. For corrected richness, precipitation was combined with temperature seasonality, while correlation of phylogenetic diversity to precipitation plus radiation indicated support for the species-energy hypothesis. Significantly high and significantly low phylogenetic diversity were found in geographically separate areas. These separate areas correlated with different climatic conditions such as seasonality in precipitation. The phylogenetic metrics identified additional areas of significant diversity, some of which have not been revealed using traditional taxonomic analyses, suggesting that different ecological and evolutionary processes have operated over the continent. Our study demonstrates that it is possible and vital to incorporate evolutionary metrics when inferring biodiversity hotspots from large compilations of data.

## Introduction

Biodiversity hotspots occur because organisms have non-random patterns of distribution, and identifying and explaining these hotspots have long been of interest to biologists, ecologists, and biogeographers (Magurran and McGill, 2011). In the past, patterns have been identified based on anecdotal observations, but with the availability of large datasets and informatic tools, these patterns can now be inferred using analytical methods. Oftentimes these analytical approaches have confirmed previous observations, however, they have also revealed new patterns, particularly at continental or global scales (Currie and Paquin, 1987; Crisp et al., 2001; Francis and Currie, 2003; Hawkins et al., 2003; Kier et al., 2005, 2009; Kreft and Jetz, 2007; Kreft et al., 2010).

The most widely used measure of biodiversity is *taxon diversity*, also known as *richness*, which is a count of the number of terminal taxa (typically species but sometimes genera or other taxonomic levels) that occur in a particular region. Richness is calculated from occurrence data with geographic locations. However, richness estimates are influenced by sampling size and effort. Therefore, a number of metrics have been developed addressing these issues in order to obtain more accurate estimates (Magurran, 2004; Maurer and McGill, 2011).

Alternatively, there are measures of biodiversity that incorporate phylogeny with geography. These phylogenetic measures can be calculated for diversity and endemism, and are advantageous because use of a phylogeny takes into account evolutionary history (Faith, 1992; Moritz and Faith, 2002; Rosauer et al., 2009). Thus, phylogenetic measures provide an estimate of how much of the evolutionary history is represented in a particular region, and is referred to as *phylogenetic diversity*. Taxon-based measures use counts of species or genera as units, but in phylogenetic measures the units are the branch lengths connecting the terminal taxa in a region. Using a species as a terminal does not add substantially more branch length than using a genus, and thus, phylogenetic measures are not particularly sensitive to the taxonomic level chosen for analysis or to splitting and lumping of taxa (Rosauer et al., 2009). Identifying regions with high diversity is critical to conservation efforts, and the inclusion of phylogenetic indices can identify areas that standard metrics do not (Forest et al., 2007; Hendry et al., 2010; Winter et al., 2013).

Key to understanding distribution patterns is how they correlate with environmental conditions. Environmental variables can be climatic, such as rainfall, radiation, and temperature, or physical, such as soil type and topography. Numerous variables have been investigated in relation to richness, but rarely for phylogenetic indices. In several cases, the variables most strongly correlated with plant richness are mean annual temperature, water availability, and evapotranspiration, as well as topography (Currie and Paquin, 1987; Francis and Currie, 2003; Hawkins et al., 2003; Kreft and Jetz, 2007; Kreft et al., 2010). Although these variables are strong predictors of richness and may be causal, historical factors could also be responsible for current distribution patterns (Qian and Ricklefs, 2004; Wiens and Donoghue, 2004). Here we explore the relationships between richness, phylogenetic diversity, and environmental variables, and address the potential role of evolutionary history through the inclusion of phylogenetic indices.

Because ferns have a broad range of habitat preferences, spanning tropical rainforests to deserts, they are widely distributed and are therefore an ideal group for understanding how diversity is distributed. Most of our understanding of fern distribution derives from transects documenting richness and its relationship to environmental variables (Dzwonko and Kornaś, 1994; Lwanga et al., 1998; Tuomisto and Poulsen, 2000; Kessler, 2002a; Aldasoro et al., 2004). Fern richness is greatest in areas of high topographic relief and complexity, high evapotranspiration, and with many rain days (Kessler, 2010). Special focus has been given to the relationship between elevational gradients and richness, with richness peaking along mid-regions of slopes (suggesting the presence of the mid-domain effect) (Kessler, 2001, 2002b, 2010; Hemp, 2002; Kromer et al., 2005; Kluge and Kessler, 2006, 2011; Watkins, 2006; Kessler et al., 2011). However, a study compiling data from multiple altitudinal transects across the globe indicates that climate (water availability and temperature) rather than the mid-domain effect has better explanatory power for species richness (Kessler et al., 2011). Furthermore, the mid-domain effect may be more relevant to particularly high elevation regions such as in the Andean tropical regions, but may be less relevant in flat and vast continents such as Australia.

Overall, there are few synthetic studies of fern diversity and their relationship to the environment over large regions; although there are studies in the Iberian Peninsula, New Zealand, and Australia, but all focusing on richness alone (Lehmann et al., 2002; Bickford and Laffan, 2006; Moreno Saiz and Lobo, 2008). At broad scales, these studies found that water availability was correlated with regions of greatest richness, and also variably identified mean annual temperature, radiation, and topography (environmental heterogeneity) as important. At these scales, it is unclear what the corresponding patterns of phylogenetic diversity are, and how they relate to the environment.

Here we examine the patterns of fern diversity using richness and phylogenetic diversity indices. In particular we test for areas of significant randomized phylogenetic diversity against a null model. We also assess the patterns of diversity observed for correlations with environmental variables. We have chosen Australia as the study area because: the continent encompasses a broad range of habitat types, the unique availability of distributional data due to the efforts of Australia's Virtual Herbarium, and a completed floristic treatment of the ferns, which means that the taxonomy has largely been standardized across states (Australian Biological Resources Study, 1998).

## Material and methods

### Geographic data

**Figure 1:**
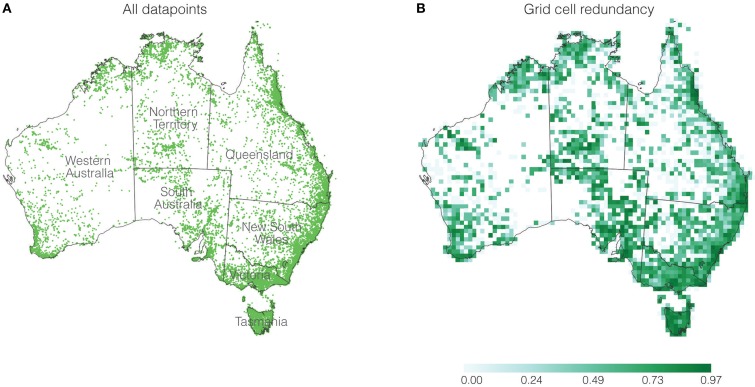
**(A)** Distribution of geographic records, and **(B)** redundancy for each grid cell. The scale bar represents redundancy values.

Records of fern collections held in Australian herbaria are available in Australia's Virtual Herbarium (AVH) (http://avh.ala.org.au/) and were downloaded for this study. The download totaled 84,134 records. Using Google Refine version 2.5 (http://code.google.com/p/google-refine/), the dataset was cleaned to remove non-fern records (e.g., algae, lycophytes, and angiosperms), foreign collections (as well as Norfolk and Macquarie Islands), cultivated material, weeds, and garden escapees. The ferns were restricted to the Classes Marattiopsida and Polypodiopsida (Smith et al., 2006), and geographic ranges were examined to determine if there were potential misidentifications for specimens found outside of their known geographic range (Australian Biological Resources Study, 1998). The taxonomy was reconciled against a classification for extant ferns (Smith et al., 2006), for a total of 386 species in 89 genera and 25 families. Misspellings in the taxonomy were also corrected. Hybrids, doubtfully identified taxa (with either cf., aff., or ?), and specimens assigned only to genus-level were discarded. Records lacking geographic coordinates were excluded from the dataset, and latitude and longitude values of the remaining records were transformed into an Albers equal area coordinate system (European Petroleum Survey Group code EPSG3577). Following all of these spatial verification and cleaning steps, the dataset consisted of 63,230 records with greatest sampling in eastern Australia (Figure 1A).

Species and genus richness were analyzed using grid cell sizes of 50 × 50 km and 100 × 100 km, which showed similar patterns (Figures S1A vs. S1B, S1C vs. S1D); thus we use the finer-scale, 50 × 50 km, cells for all of the analyses presented in order to minimize aggregation of the environmental data. Inspection of species vs. genus richness (Figures S1A vs. S1C) show that the patterns obtained are congruent. Therefore, the genus level results are presented herein because the phylogenetic diversity analyses were conducted at the genus level as there are currently inadequate molecular data to generate species-level phylogenies. A general relationship between species richness and higher taxon richness is found in many other studies as well (Williams and Gaston, 1994; Williams et al., 1997; Aldasoro et al., 2004; Currie and Francis, 2004).

Using the 50 × 50 km grid cell size resulted in a total of 1986 grid cells across Australia with at least one fern record. When the data were aggregated within the grid cells, the 63,230 records were reduced to 18,050 unique occurrences. As an example, the best-collected genus *Cheilanthes* comprised 6466 records but after aggregation into the 1986 grid cells there were 1320 unique occurrences. As the calculations only take into account occurrence in a cell, and not abundance values, there are multiple redundant records of the same taxon in each grid cell (Figure 1B). Redundancy, a measure of sampling quality, is scaled from zero to one (Garcillán and Ezcurra, 2003; Laffan et al., 2010). Redundancy values close to zero indicate possible under-sampling, while those close to one indicate well-sampled cells. Redundancy shows some variability across the continent, most closely linked to regions of population density (Figure 1B). There are some regions with values closer to zero, but since ferns are rarely found in these arid regions, the lack of duplicate sampling is likely not influencing the patterns we observe.

### Molecular data and phylogenetic analyses

All of the available sequences for each genus were downloaded from Genbank, regardless of species or geographic origin. Initially seven chloroplast markers were assessed for potential use in the phylogenetic analyses, but of these only three markers (*atpA*, *atpB*, and *rbcL*) were selected because they had the lowest amount of missing data. There were 420 sequences for *atpA*, 1117 sequences for *atpB*, and 2454 sequences for *rbcL*; the sequences were aligned using MAFFT version 6.860b (Katoh and Toh, 2008). Using each of these markers separately, a maximum likelihood phylogeny was constructed using GARLI (Genetic Algorithm for Rapid Likelihood Inference) version 0.951 (Zwickl, 2006), and set to terminate automatically using default parameters. In order to select one representative sequence for each genus, each of the three phylogenies was examined to determine that the sequences representing a genus were monophyletic. In cases where a genus was paraphyletic, this was typically due to an outlier sequence; thus, the phylogenetic position of the genus was checked against published phylogenies to verify that the sequence was indeed an outlier, and then it was discarded. Subsequently, two additional criteria were applied to select a representative sequence: first, a sequence was obtained from an Australian species, and second, if there were no Australian species, another species was selected if all three markers were available (or otherwise two markers).

New sequences were generated for seven genera not previously represented in Genbank, with GenBank accession numbers KP164480-KP164497 (Table S1). The following primers were used as amplification and sequencing primers for *rbcL*: ESRBCL1F, ESRBCL628F, ESRBCL654R, ESRBCL1361R; *atpB*: ESATPB172F, ESATPE45R; and *atpA*: ESATPF412F, ESATPF412F, ESTRNR46F, ESTRNR46F (the latter two as reverse primers) (Schuettpelz et al., 2006; Schuettpelz and Pryer, 2007). In addition, sequencing only primers were used for *atpB*: ATPB1163F, ATPB910R; and *atpA*: ESATPA856F, ESATPA877R (Schuettpelz et al., 2006; Schuettpelz and Pryer, 2007). PCR amplification was performed at 95°C for 10 min; 30 cycles of 94°C for 30 s, 64°C for 1 min, and 72°C for 45 s; and one cycle at 72°C for 5 min.

One representative sequence per marker for each of the 89 genera was assembled into a matrix for a total of 3893 base pairs. In this matrix, *atpA* and *atpB* were each missing sequences for four genera, and *rbcL* was complete. In this final matrix, the genus *Actinostachys* was incomplete at two of the three markers, *Anogramma, Colysis*, *Cyclosorus*, and *Marattia* were incomplete at one of three markers, and all other genera were complete for all three markers (Table S1). Molecular phylogenetic analysis of the partitioned, concatenated three markers were conducted using RAxML-HPC 7.3.2 using the CIPRES online portal (Miller et al., 2010). The supermatrix and resultant trees are deposited in TreeBase as study #15499. Overall, there was strong support for most of the nodes in the phylogeny (Figure S2), and the phylogenetic relationships were verified against a tree derived from the most well sampled and complete molecular dataset of ferns to date (Schuettpelz and Pryer, 2007).

To assess the impact of taxonomic changes on the results, we compared the PD values from this study to the PD values of an updated tree. In this updated tree, multiple recent taxonomic changes were incorporated: *Doodia* and *Pteridoblechnum* were omitted because they were synonymized into *Blechnum* (Perrie et al., 2014); the new genus *Telmatoblechnum*, which is a segregate of *Blechnum*, was included (Perrie et al., 2014); *Oenotrichia* and *Coveniella* were omitted because they had been synonymized into *Lastreopsis* (Labiak et al., 2014a,b); the new genus *Parapolystichum*, which is a segregate of *Lastreopsis*, was added (Labiak et al., 2014a,b); and *Revwattsia* was removed because it was synonymized into *Dryopteris* (McKeown et al., 2012). When we reanalyzed the data with the new tree, the range of PD values did not change significantly. In fact, when we subtracted the original PD value from the new PD value (calculated within the same grid cell) the differences ranged from −0.00000050 to 0.00000050, and the mean difference across all 1986 grid cells was −0.00000006. As a comparison, the PD values in this study were 0.01879–0.9308. It is clear, therefore, that recent taxonomic changes have little impact on the results.

### Analysis of diversity

The phylogeny derived from the three markers and the geographic data were imported into Biodiverse version 0.17 (Laffan et al., 2010). Several measures of diversity were calculated: species richness (SR), genus richness (GR), Margalef genus richness (MR), phylogenetic diversity (PD), and randomized phylogenetic diversity (PD_rand_).

The richness measures (SR and GR) are a direct count of the number of taxa occurring in each grid cell. To correct for uneven sampling effort among grid cells, the Margalef diversity metric is used. This metric standardizes richness across grid cells by dividing richness (*R* - 1) by the natural log of the number of samples (N) in a grid cell, thus *R*_Margalef_ = (*R* − 1)/ln N (Magurran, 2004; Maurer and McGill, 2011).

Phylogenetic diversity (PD) measures the amount of the phylogenetic tree that is represented in a grid cell (Faith, 1992), which represents the sum of branch lengths in that grid cell. Specifically, this measure takes all of the terminal taxa that are found in one grid cell and sums the branches connecting them along a path to and including the root node. We depict PD as a proportion of the total tree length. PD is expected to be correlated with richness since with more terminal taxa there are more branch lengths to be summed (Tucker and Cadotte, 2013)—thus in rich areas the PD is expected to be larger. Indeed, we found that the GR and PD values within each grid cell are highly correlated; the generalized linear model (GLM) yielded an *r*^2^ equivalent of 0.895.

To test for statistical significance of the PD results, we used a randomization test (Mishler et al., 2014). First, all of the 18,050 unique occurrences (see “Geographic Data”) are pooled, and records from this pool are randomly assigned (without replacement) to the grid cells based on a constraint that the number of unique occurrences per grid cell are kept constant. This has the effect of keeping the number of terminal taxa per cell constant, for example, if a grid cell had 11 unique occurrences, then 11 unique occurrences from the pool were randomly assigned to that grid cell. In addition, the range size of each terminal taxon was kept constant. Second, PD_rand_ is recalculated for each grid cell using the randomly assigned unique occurrences and the original phylogeny. These two steps yield new PD_rand_ values for each grid cell. This process is repeated 999 times to give a distribution of PD_rand_ values, and the original PD_rand_ value is compared to the 999 PD_rand_ values. If the original PD falls in the upper or lower 2.5% of the 1000 values, the PD_rand_ of that cell is judged statistically significant. This method is similar to a randomly generated null community, known as null model 1 in the software Phylocom (Webb et al., 2008), or the null constrained model (Kembel and Hubbell, 2006). We recognize that this is one of several possible null models (Gotelli, 2000), each of which tests different questions, and is associated with different assumptions, but this model is appropriate for our objective to identify cells with significantly high or low PD values. Furthermore, we conducted an additional analysis where we standardized the PD value by the number of taxa (PD/richness), referred to as relative phylogenetic diversity (PD_rel_) by Davies et al. (2007). The PD_rel_ randomization results are identical to the PD randomizations, indicating the robustness of the metrics; herein we refer to the PD randomizations only.

### Environmental predictors of diversity

Eleven environmental variables were assessed against the diversity indices. These variables encompass temperature, precipitation, topography and substrates (Table 1). The 11 variables were selected because they are “independent” and representative of the predominant conditions. The climatic data were from selected BIOCLIM layers described and developed as part of ANUCLIM version 5.1 (Hutchinson et al., 2000). The soil layers were obtained from a database that was generated as part of a national survey (National Land and Water Resources Audit, 2002).

**Table 1:** **Explanation of environmental variables used in this study, see Methodsd**.

**Environmental variable**	**Description**
Annual precipitation (BIOCLIM 12)	Monthly precipitation estimates (mm)
Annual mean temperature (BIOCLIM 1)	The mean of the week's maximum and minimum temperature (°C)
Annual mean radiation (BIOCLIM 20)	The mean of all the weekly radiation estimates (Mj/m^2^/day)
Precipitation of coldest quarter (BIOCLIM 19)	Total precipitation over the coldest period of the year
Radiation seasonality (BIOCLIM 23)	Standard deviation of the weekly radiation estimates expressed as a percentage of the annual mean (Mj/m^2^/day)
Precipitation seasonality (BIOCLIM 15)	Standard deviation of the weekly precipitation estimates expressed as a percentage of the annual mean (mm)
Temperature seasonality (BIOCLIM 4)	Standard deviation of the weekly mean temperatures estimates expressed as a percentage of the annual mean (°C)
Ridge top flatness	Metric of the topographic flatness derived from a surface of 9 s grid cells (dimensionless); higher values identify high flat areas while low values indicate low steep areas.
Rock grain size	Lithological property of the bedrocks related to the mean grain size (0–10 units)
Sand	Content of sand on the top 30 cm soil layer estimated from soil maps at a resolution of 1 km (%)
Clay	Content of clay on the top 30 cm soil layer estimated from soil maps at a resolution of 1 km (%)

The predictors were tested against all of the indices as single variables (e.g., mean annual temperature). The top five single variables (based on the Akaike Information Criterion, AIC, see below) were examined further as interaction variables (e.g., mean annual temperature × topography), and as additive variables (e.g., mean annual temperature + topography). Some of the grid cells did not have environmental data and were excluded from the analyses, leaving 1913 grid cells in this part of the study. Most of these points were on the coast.

#### Generalized linear models

Because the indices do not have a normal distribution, standard linear models (LM) were not appropriate for analyzing their relationship to the 11 environmental variables. Based on examination of the data, a Poisson distribution best described the GR data, and a gamma distribution was most appropriate for the PD data. Consequently, generalized linear models (GLMs) were used because they allow for data that are not normally distributed, and a GLM was calculated for each variable and diversity metric with their appropriate distributions. A different approach was required for PD_rand_ results since they are either significant or not, that is *p* ≤ 0.05. The statistically significant values were transformed into 1 and all other values were set to 0. These transformed data were then analyzed using GLMs with binomial distributions; the binomial is the only distribution that accepts categorical data, whereas all other distributions require continuous data. The higher and lower than expected PD_rand_ were analyzed separately (high PD_rand_ = 1 or low PD_rand_ = 1). In total there were 40 high PD_rand_ values and 84 low PD_rand_ values. For each GLM, the following were recorded: AIC, percent of deviance explained (equivalent to *r*^2^), the statistical significance of the z-score of Wald's test, and whether the slope of the line was positive or negative. AIC values within 3 units of the best model were considered equally informative.

#### Detecting spatial autocorrelation using Moran's I

To test whether there are biases due to spatial autocorrelation, Moran's I was calculated on the residuals of each standard linear model (LM). Spatial autocorrelation can yield misleading results because points that are close to each other will tend to share the same taxa, and are not independent (Dormann et al., 2007). Testing for spatial autocorrelation required a series of steps.

Firstly, a matrix was created that establishes which points are neighbors (referred to as a neighbors list), thus identifying the adjacent grid cells that could be affected by spatial autocorrelation. A distance criterion based on grid cell size was used to define the nearest neighbors. Since the grid cells were 50 × 50 km, all of the eight adjoining grids are in the radius of 75 km and so this latter value was set as the distance value for defining the nearest neighbors. Of the 1913 grid cells, 18 cells did not have neighbors within 75 km, and were subsequently excluded from the analyses (Figure S3). For the PD_rand_ data, neighbors were defined at either 150 or 300 km because the radius of 75 km resulted in too few neighbors to draw any meaningful conclusions. There were 40 PD_rand_ high values, and when using the smaller radius size, 15 of these points were deleted because there were no neighbors. In contrast, using the larger radius size required deletion of only four neighbors (Figures S4A,B). For the PD_rand_ low analyses there were 84 values, and at the 150 km radius nine points were removed, while four were removed at the 300 km level (Figures S4C,D).

Secondly, weights were assigned to the neighbor relationships (via a spatial weights matrix). All of the relationships were given equal values of 1. Finally, the Moran's I global tests were run using the spatial weights matrix and a LM for each of the environmental variables and each of the indices. Moran's I values around zero indicate that there is no spatial autocorrelation in the residuals of the LMs and so neighbors have random values that are not linked. Moran's I values toward 1 indicate a positive correlation where adjoining grid cells are likely to share the same value (high-high; low-low); values toward -1 indicate a negative correlation suggesting that neighbors are more likely to have opposite values (low-high). The *p*-values of the Moran's I results were also recorded.

#### Accounting for spatial autocorrelation using spatial autoregressive models

To account for spatial autocorrelation, spatial autoregressive (SAR) models were used (Dormann et al., 2007; Kissling and Carl, 2007). There are three types of SARs, each of which account for autocorrelation via the addition of an extra term, a covariance matrix, to a standard linear model (LM). (1) SAR_lag_ is the lagged response model, which accounts for spatial autocorrelation by adding a term for spatial autocorrelation in the response variable (in this study, the richness indices), (2) SAR_mix_ is the lagged mixed model accounting for autocorrelations in both the indices and the environmental variables (the response and predictor variables), and (3) SAR_err_ is a spatial error model that accounts for autocorrelation via an error term (i.e., in neither the indices nor the variables) (Dormann et al., 2007). For our data, the three SAR models (SAR_err_, SAR_lag_, and SAR_mix_) identified the same variables as the best models, however, SAR_mix_ occasionally yielded some differences from the other two models (not shown). Given the high error rate in SAR_mix_ identified in earlier studies, this result is not surprising and is likely to be a type I error (Dormann et al., 2007; Kissling and Carl, 2007). Tests of all three models indicate that SAR_err_ has the least bias and error among all the SAR models (Kissling and Carl, 2007). Therefore, we present the results of SAR_err_ here.

In the same steps as the calculations of the Moran's I, a neighbor list was constructed, followed by a spatial weights matrix; both were identical to that used for calculating the Moran's I. The SARs were then calculated using a LM for each of the environmental variables and each of the indices, together with the spatial weights matrix and the extra autocorrelation term. AICs were calculated for each model to determine which of the environmental variables best fitted each of the indices. The significance value for each SAR model was also recorded. SARs were calculated for all of the indices except for PD_rand_, which are statistical significance values and are therefore not appropriate for analysis using a LM.

The GLMs, Moran's I, and the SARs were calculated using the spdep package version 0.5–56 (Bivand, 2014) in R version 2.15.3 (R Core Team, 2014).

## Results

### Richness

**Figure 2:**
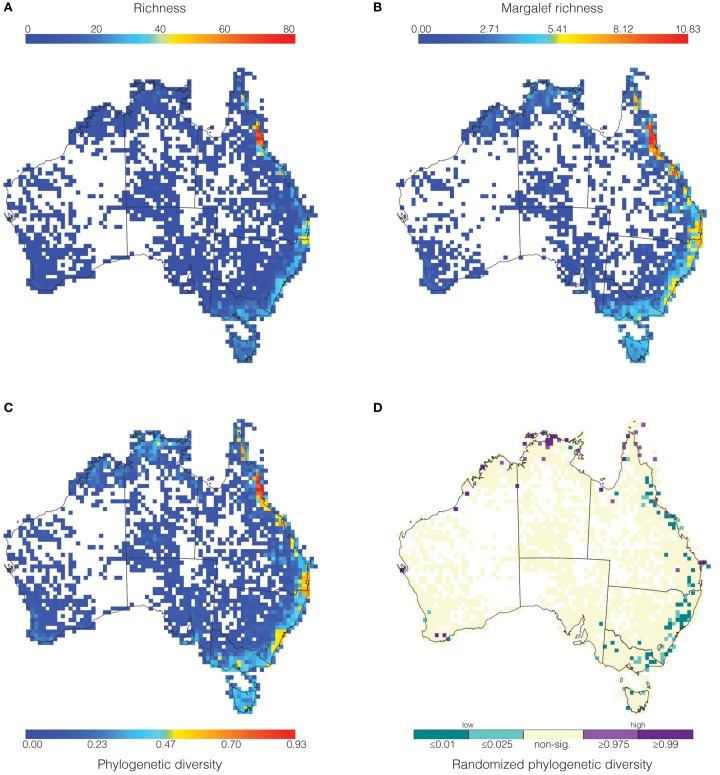
**Maps showing grid cells values for (A) richness, (B) Margalef richness, (C) phylogenetic diversity (as a proportion of total phylogenetic diversity), and (D) significant phylogenetic diversity identified via randomization**.

The majority of fern richness is found along the eastern coast in Australia (Figure 2A). The greatest richness occurs in the Wet Tropics in Queensland with 80 genera (red-orange, Figure 2A), while the Border Ranges (between Queensland and New South Wales) is the second richest area with approximately 45 genera (yellow, Figure 2A). There is a continuous tract of low richness (approximately 30–40 genera, light blue Figure 2A) extending from Tasmania along the east coast of the continent. However, the vast majority of Australia has poor richness (less than 10 genera, dark blue, Figure 2A). Correcting for sampling using Margalef richness (MR) (Figure 2B) intensifies the patterns observed in uncorrected richness (Figure 2A). Namely, the east coast tract of low richness increases to medium richness with patches of medium-high richness. These patches correspond to the Border Ranges and Sydney Sandstone regions.

### Phylogenetic diversity

Phylogenetic diversity (PD) (Figure 2C) shows similar patterns to richness (Figure 2A), but most especially to MR (Figure 2B). In agreement with the two richness metrics, PD also shows the Wet Tropics as the greatest hotspot with 90% (0.90) of the phylogeny represented. Notable similarities between MR and PD are the medium-high values in the Border Ranges and the Sydney Sandstone regions, with about 50–60% of the tree present for PD (Figure 2C). Also, the Northern Territory and Tasmania have corresponding regions of low MR and low PD (Figures 2B,C light blue). As for the richness metrics, the overwhelming pattern is that most of the continent has poor PD (dark blue, Figure 2C), and that the east coast hosts most of the fern PD (light blue-yellow-orange-red, Figure 2C).

### Statistically significant phylogenetic diversity

The phylogenetic diversity randomizations showed that cells with significantly high PD_rand_ were mostly separate from those with low PD_rand_ (Figure 2D). The high PD_rand_ regions are concentrated toward the north of Australia, in the most northern parts of the Northern Territory and Queensland (purples, Figure 2D), whereas the low PD_rand_ regions are aggregated along the east coast (teals, Figure 2D). We note that PD_rand_ and PD standardized by richness (not shown) yielded identical results, indicating that PD_rand_ is not sensitive to sampling bias.

The significantly high PD_rand_ cells are characterized by the overrepresentation of taxa with long branches representing disparate clades of the phylogeny. Principally these are the combination of a long-branched early diverging lineage (Schizaeales) and a long-branched derived family, Pteridaceae. The genus *Lindsaea* is typically found in these cells, and in some cells the early diverging lineages Salviniales and Gleichneiales are present too. Alternatively, significantly high PD_rand_ cells can have one taxon representing a particularly long branch, either *Acrostichum* or *Ceratopteris*. Conversely, the significantly low PD_rand_ cells can have one taxon with an especially short branch, for example *Abrodictyum* in the early diverging Hymenophyllaceae. Examination of the taxonomic composition of the cells that have significantly low PD_rand_ reveals three additional ways in which low PD_rand_ can arise. First, there are few taxa in that cell, and they are all restricted to one family, such as Pteridaceae. Second, there is a moderate number of taxa but they are restricted to one clade, the tree fern + polypod clade, with the early diverging lineages not represented. Thirdly, again there is moderate to high numbers of taxa, but here the early diverging lineages are represented, while the eupolypods I (composed of Nephrolepidaceae, Lomariopsidaceae and Polypodiaceae) are missing.

### Predictors

When the indices were correlated with the environmental variables, the GLMs for richness and PD indicated that annual precipitation together with mean annual radiation, as additive or interactive variables, is the best predictor (Table 2). In the case of Margalef richness, the GLM with the lowest AIC was annual precipitation together with seasonality in temperature. However, Moran's I values from 0.48 to 0.59 show that these results are biased by spatial autocorrelation (Table 2). When spatial autocorrelation is taken into account using SARs, the best predictor for richness changes to annual precipitation by topography (ridge top). For both Margalef richness and PD, the models recovered using GLM and SAR are identical. All of the best performing models are explained by a positive interaction, and all included a term for annual precipitation, indicating the importance of water in determining the diversity of ferns (Table 2). Regardless of the metric used, the two-factor models outperformed all of the single-factor models.

**Table 2:** **Statistical relationships between environmental variables and the richness, Margalef richness and phylogenetic diversity metrics**.

	**Richness**							**Margalef richness**							**Phylogenetic diversity**						
	**SAR**		**GLM**					**SAR**		**GLM**					**SAR**		**GLM**				
	**ΔAIC**	***z***	**ΔAIC**	**DE%**	***p***	**slope**	***I***	**ΔAIC**	***z***	**ΔAIC**	**DE%**	***p***	**slope**	***I***	**ΔAIC**	***z***	**ΔAIC**	**DE%**	***p***	**slope**	***I***
Ann precip × Topographic flatness	0	^***^	1589	52.49	ns	+	0.55	33	^***^	146	51.95	^***^	−	0.56	21	^***^	189	57.18	^***^	−	0.46
Ann precip × Ann mean radiation	16	^***^	0	62.28	^***^	+	0.59	31	^*^	23	56.46	^***^	+	0.60	0	^***^	0	61.10	^*^	+	0.48
Ann precip × Temp seasonality	18	^***^	558	58.84	^***^	+	0.59	0	^**^	0	57.27	^***^	+	0.56	76	^***^	173	57.52	^***^	+	0.49
Ann precip + Ann mean radiation	38	^***^	1443	53.38	^***^	−	0.60	36.1	^***^	40	55.83	^***^	−	0.60	0.3	^***^	2	61.03	ns	−	0.29
Ann precip + Temp seasonality	86	^***^	2656	45.91	ns	+	0.61	98	^***^	205	49.55	^*^	+	0.61	134	^***^	307	54.50	ns	+	0.36
Ann precip × Precip of coldest quarter	89	^***^	594	58.62	^***^	−	0.61	75	^***^	69	54.85	^***^	−	0.63	47	^***^	38	60.34	^***^	−	0.50
Ann precip + Precip of coldest quarter	89	^***^	2416	47.39	^***^	+	0.61	110	^***^	150	51.73	^***^	+	0.64	69	^***^	129	58.42	ns	+	0.32
Ann precip + Topographic flatness	177	^***^	1587	52.49	^***^	−	0.62	104	^***^	181	50.51	^***^	−	0.61	147	^***^	246	55.87	ns	−	0.32
Ann precip	230	^***^	2655	45.90	^***^	+	0.64	146	^***^	210	49.30	^***^	+	0.65	198	^***^	310	54.37	^***^	+	0.53
Ann mean radiation × Temp seasonality	455	^***^	2649	45.96	^***^	−	0.67	373	^***^	455	38.61	^***^	+	0.72	481	^***^	495	50.00	^**^	+	0.59
Ann mean radiation + Temp seasonality	497	^***^	2984	43.88	^***^	−	0.68	374	^***^	384	41.84	^***^	−	0.72	510	^***^	500	49.81	ns	−	0.37
Temp seasonality + Precip of coldest quarter	531	^***^	4069	37.20	^***^	+	0.69	438	^***^	476	37.44	^***^	+	0.75	550	^***^	576	47.85	ns	+	0.40
Temp seasonality × Precip of coldest quarter	532	^*^	3657	39.75	^***^	+	0.69	439	^***^	456	38.52	^***^	+	0.75	552	^***^	560	48.33	^***^	+	0.61
Ann mean radiation × Topographic flatness	549	^***^	3902	38.24	^***^	+	0.69	505	^***^	732	23.65	ns	+	0.73	749	^***^	1039	34.24	^***^	+	0.66
Precip of coldest quarter × Topographic flatness	611	^***^	5033	31.28	^***^	−	0.70	484	^***^	579	32.30	^**^	+	0.71	833	^***^	1197	28.87	^***^	−	0.66
Ann mean radiation + Topographic flatness	634	^***^	3928	38.07	^***^	−	0.71	532	^***^	731	23.59	^***^	+	0.75	811	^***^	1078	32.91	ns	−	0.41
Ann mean radiation + Precip of coldest quarter	667	^***^	4909	32.03	^***^	−	0.74	563	^***^	799	19.47	^**^	+	0.77	847	^***^	1150	30.44	ns	−	0.46
Ann mean radiation × Precip of coldest quarter	669	^***^	4497	34.58	^***^	+	0.74	563	^***^	738	23.31	^*^	+	0.77	849	^***^	1151	30.46	ns	+	0.70
Ann mean radiation	676	^***^	4928	31.90	^***^	−	0.73	562	^***^	811	18.60	^***^	+	0.78	852	^***^	1148	30.44	^***^	−	0.70
Precip of coldest quarter + Topographic flatness	715	^***^	5093	30.89	^***^	−	0.73	608	^***^	817	18.29	^***^	+	0.76	917	^***^	1269	26.19	ns	−	0.44
Temp seasonality × Topographic flatness	725	^***^	4028	37.47	^***^	+	0.68	563	^***^	819	18.33	ns	+	0.74	756	^***^	865	39.75	^***^	+	0.61
Precip of coldest quarter	776	^***^	6684	21.08	^***^	+	0.76	652	^***^	936	10.34	^***^	−	0.80	979	^***^	1367	22.42	^***^	+	0.72
Temp seasonality + Topographic flatness	788	^***^	4073	37.18	^***^	−	0.70	510	^***^	588	31.71	^***^	+	0.72	815	^***^	925	37.85	ns	−	0.42
Temp seasonality	890	^***^	6504	22.19	^***^	−	0.74	603	^***^	809	18.69	^***^	+	0.80	920	^***^	1087	32.53	^***^	−	0.67
Topographic flatness	1066	^**^	7429	16.49	^***^	−	0.75	739	ns	908	12.20	^***^	+	0.76	1272	^**^	1681	9.39	^***^	−	0.71
Ann mean temp	1071	^***^	8575	9.43	^***^	−	0.78	786	ns	1033	3.33	^***^	+	0.80	1301	^***^	1767	5.51	^***^	−	0.75
Radiation seasonality	1158	^***^	9751	2.19	^***^	+	0.79	816	ns	1074	0.28	ns	−	0.81	1374	^***^	1863	0.92	^**^	+	0.76
Clay	1172	^**^	9959	0.91	^***^	−	0.79	818	^***^	1075	0.17	ns	+	0.81	1357	^***^	1818	3.08	^***^	−	0.75
Precip seasonality	1175	^**^	9985	0.74	^***^	−	0.79	819	ns	1077	0.02	ns	+	0.81	1388	^**^	1881	0.07	ns	−	0.76
Sand	1177	^**^	10,039	0.41	^***^	+	0.79	818	^***^	1077	0.08	ns	−	0.81	1367	^***^	1842	1.94	^***^	+	0.76
Rock grain size	1181	ns	10,089	0.11	^***^	−	0.79	810	ns	1068	0.77	^**^	+	0.80	1389	ns	1882	0.00	ns	−	0.76

SAR, simultaneous autoregressive model, calculated using the spatial error model; GLM, generalized linear model; ΔAIC, Akaike information criterion (AIC) value of a given model relative to best model in the set; DE%, percentage of the deviance explained; I, Moran's I of residuals of linear model, note that all Moran's I are highly significant ≤ 0.001. Significance measures are reported as z- and p-values. The z-score is given for the simultaneous autoregressive models, and the p-value is given for the generalized linear model. Abbreviations for significance levels:

* ≤ 0.05;

** ≤ 0.01;

*** *≤ 0.001; ns, not significant. See Table 1 for environmental variables; “+” indicates additive model of the two variables, and “×” indicates an interactive model for the two variables. Degrees of freedom are provided in **Table 3***.

The randomizations of PD are explained by a different set of environmental factors compared to the other indices (Table 3). In addition, high PD_rand_ and low PD_rand_ are each explained by different environmental factors. The high PD_rand_ areas correlate with mean annual radiation added/by temperature seasonality (0 compared to 2 ΔAIC respectively, Table 3), and a close model was temperature seasonality plus precipitation in the coldest quarter (3 ΔAIC, Table 3). Overall, the highest performing models for high PD_rand_ all had temperature seasonality in common. The low PD_rand_ areas also correlate with temperature seasonality, but by annual precipitation. For 150 or 300 km values at which neighbors were defined, Moran's I values were all close to zero indicating that spatial autocorrelation was not present in these datasets (Table 3).

**Table 3:** **Statistical relationships between environmental variables and PD_rand_, randomized phylogenetic diversity, with higher (high PD_rand_) or lower (low PD_rand_) than expected PD for statistically significant cells only**.

	**Randomized PD high**						**Randomized PD low**						***df***
	**GLM**						**GLM**						
	**ΔAIC**	**DE%**	***p***	**slope**	**I 300K**	**I 150K**	**ΔAIC**	**DE%**	***p***	**slope**	**I 300K**	**I 150K**	
Ann mean radiation + Temp seasonality	0	29.70	^***^	−	−0.08	−0.11	74	12.46	ns	−	−0.04	−0.04	3
Ann mean radiation × Temp seasonality	2	29.71	ns	−	−0.10	−0.11	42	17.41	^***^	−	−0.04	−0.03	4
Temp seasonality + Precip of coldest quarter	3	28.99	ns	−	−0.06	−0.10	106	7.86	^***^	+	−0.03	−0.03	3
Temp seasonality	4	28.12	^***^	−	−0.05	−0.08	144	2.09	^***^	−	−0.01	−0.01	2
Temp seasonality × Precip of coldest quarter	4	29.11	ns	+	−0.10	−0.10	82	11.62	^***^	+	−0.04	−0.02	4
Temp seasonality + Topographic flatness	5	28.38	ns	−	−0.07	−0.10	67	13.45	^***^	−	−0.01	−0.01	3
Temp seasonality × Topographic flatness	5	28.81	ns	+	−0.07	−0.11	64	14.18	*	−	−0.02	−0.01	4
Ann precip + Temp seasonality	6	28.17	^***^	−	−0.09	−0.09	99	8.87	^**^	+	0.00	−0.01	3
Ann precip × Temp seasonality	7	28.46	ns	−	−0.10	−0.12	0	23.51	^***^	+	0.00	0.00	4
Ann precip × Precip of coldest quarter	14	26.52	*	−	−0.11	−0.11	66	13.98	^***^	−	−0.04	−0.05	4
Ann precip × Ann mean radiation	17	25.89	^***^	+	−0.11	−0.12	43	17.27	^***^	+	−0.05	−0.05	4
Ann precip + Precip of coldest quarter	22	24.17	^***^	−	−0.09	−0.12	92	9.91	^***^	+	−0.04	−0.05	3
Ann precip + Ann mean radiation	30	22.00	^***^	+	−0.09	−0.12	63	14.02	^***^	−	−0.05	−0.05	3
Ann mean temp	48	16.86	^***^	+	−0.05	−0.06	88	10.20	^***^	−	−0.06	−0.05	2
Precip seasonality	53	15.44	^***^	+	−0.04	−0.06	144	1.97	^***^	−	−0.04	−0.04	2
Radiation seasonality	57	14.57	^***^	−	−0.05	−0.06	143	2.20	^***^	+	−0.04	−0.04	2
Ann precip × Topographic flatness	59	15.01	ns	+	−0.09	−0.11	52	15.95	ns	+	−0.01	0.00	4
Ann precip	59	13.88	^***^	+	−0.07	−0.10	104	7.87	^***^	+	0.00	0.00	2
Ann precip + Topographic flatness	60	14.32	ns	+	−0.09	−0.12	51	15.83	^***^	−	0.00	−0.01	3
Ann mean radiation × Precip of coldest quarter	89	7.32	ns	−	−0.10	−0.10	58	15.12	^***^	+	−0.05	−0.04	4
Ann mean radiation + Precip of coldest quarter	89	6.77	^***^	−	−0.09	−0.10	73	12.65	ns	−	−0.04	−0.04	3
Precip of coldest quarter	96	4.37	^**^	−	−0.06	−0.06	110	7.05	^***^	+	−0.03	−0.03	2
Precip of coldest quarter + Topographic flatness	98	4.48	ns	−	−0.07	−0.09	53	15.60	^***^	−	−0.03	−0.03	3
Precip of coldest quarter × Topographic flatness	99	4.75	ns	−	−0.09	−0.11	55	15.60	ns	+	−0.03	−0.03	4
Sand	110	0.98	ns	+	−0.02	−0.03	156	0.37	ns	−	−0.03	−0.02	2
Ann mean radiation	110	0.88	ns	+	−0.08	−0.08	73	12.28	^***^	−	−0.04	−0.04	2
Clay	110	0.80	ns	−	−0.04	−0.05	158	0.07	ns	+	−0.03	−0.02	2
Ann mean radiation + Topographic flatness	112	0.92	ns	−	−0.09	−0.10	34	18.31	^***^	−	−0.04	−0.04	3
Ann mean radiation × Topographic flatness	113	1.14	ns	−	−0.08	−0.10	34	18.55	ns	−	−0.05	−0.05	4
Rock grain size	113	0.09	ns	+	−0.02	−0.08	156	0.31	ns	−	0.00	0.01	2
Topographic flatness	113	0.00	ns	+	−0.07	−0.09	77	11.82	^***^	−	0.00	0.00	2

*See Table 2 for abbreviations and full explanation*.

## Discussion

### Comparing diversity metrics and hotspots

Using the largest fern dataset assembled to date, we find that the Wet Tropics is the most diverse region identified using three different diversity metrics (red cells in Figures 2A–C). When richness is compared to PD there are regions that have greater phylogenetic diversity than richness. However, when uneven sampling effort is accounted for, PD and richness (measured as Margalef richness) are generally in agreement. Additional regions with significant diversity are the Border Ranges and Sydney Sandstone, both in coastal eastern Australia (light blue in Figure 2A vs. orange-yellow in Figure 2C), and Kakadu–Alligator Rivers in the Northern Territory, the Kimberly region in northwest Western Australia, southwest Western Australia, and Tasmania (dark blue in Figure 2A vs. light blue-yellow in Figure 2C).

The disparity between uncorrected richness and phylogenetic diversity indicates that richness is not necessarily predictive of phylogenetic diversity. Studies elsewhere have shown a disparity between richness and phylogenetic diversity, sometimes when sampling has been accounted for (Davies et al., 2007; Forest et al., 2007; Huang et al., 2011). Such disparity emphasizes that caution is needed when using richness alone as a metric of diversity. Thus, we focus on Margalef richness results because the sampling bias has been corrected. From a conservation viewpoint, diversity must be assessed using sample standardization for richness as well as using phylogenetic diversity. Regions with high phylogenetic diversity can harbor unrecognized diversity value (Moritz and Faith, 2002; Rosauer and Mooers, 2013), and may be culturally and medicinally useful too (Forest et al., 2007).

In an earlier study, fern richness across Australia was documented at the species scale and using a dataset approximately half the size of the present study (Bickford and Laffan, 2006). Differences in the datasets are most evident in the increased sampling in inland regions. Regardless of the dataset size (Figure S1A our study, vs. Figure 2A, Bickford and Laffan, 2006), or the taxonomic level (Figure 1A our study vs. Figure S1A our study), there remains greatest richness along the east coast of Australia with a substantial hotspot in the Wet Tropics. This Wet Tropics hotspot was earlier detected for a dataset of vascular plants, comprising principally of angiosperms (Crisp et al., 2001). However, other significant angiosperm hotspots, including the significant southwest Western Australia hotspot, are not shared with ferns (Crisp et al., 2001; González-Orozco et al., 2011, 2014; Schmidt-Lebuhn et al., 2012; Kooyman et al., 2013); this is not surprising given the more arid conditions in these latter hotspots, as well as the dependence on water for ferns during the reproductive phase of their life cycle, and preference for moist conditions. The inconsistency among hotspots of various floristic groups indicates that hotspots need to be inferred on a case-by-case basis. These may be the result of the dissimilar ecological preferences as well as different diversification histories. Interestingly, two angiosperm hotspots, the Border Ranges and Sydney Sandstone, are only observed as fern hotspots when using Margelef richness and phylogenetic diversity. On the other hand, liverworts and mosses show greatest diversity along the east coast (Stevenson et al., 2012), largely matching the pattern seen in the ferns (Nagalingum et al., 2014). These corresponding patterns likely reflect the more critical requirement for water of all of these seed-free plant groups, and its greater availability in these regions.

### Explaining the distribution of diversity

Differences in diversity distribution have been attributed to a variety of mechanisms. In ferns, the overwhelming pattern is the near-absence of diversity in the central arid interior of the continent, and a hotspot in the Wet Tropics. The Wet Tropics likely represents the ancestral niche for ferns, whereas survival in arid biomes require a suite of adaptations that have arisen in only two of the 89 genera examined here. This pattern fits the ancestral niche hypothesis that predicts that greater diversity will be present in an ancestral niche because more taxa have accumulated here as the group has remained in that niche; at the same time, the group is unable to disperse to other niches without the evolution of suitable adaptations (Crisp et al., 2009; Wiens et al., 2010). In addition, a range of historical factors has been used to explain the previously observed greater diversity in the tropics. These range from elevated speciation rates and decreased extinction rates (compared to extra-tropical regions; the cradle-museum hypothesis), a greater geographical extent of tropical rainforests in the past (the area effect), and an older age of the tropics (the age effect) (Qian and Ricklefs, 2004; Wiens and Donoghue, 2004). Although we were not able to conduct a detailed examination of traits and historical factors with the present dataset, it is likely that both have shaped the distribution patterns we observed for the ferns.

Biodiversity hotspots and underlying differences in diversity distribution are commonly linked to current climatic and environmental conditions—although this has led to discussion between the role of current versus historical factors (Currie and Paquin, 1987; Latham and Ricklefs, 1993; Qian and Ricklefs, 2000; Francis and Currie, 2003; Ricklefs, 2004). Regardless of the scale, from global modeling studies (Kreft and Jetz, 2007; Kreft et al., 2010) to regional meta-analyses (Lehmann et al., 2002; Bickford and Laffan, 2006), plot-based elevational transects (Kessler, 2000, 2001; Bhattarai et al., 2004; Grytnes and Beaman, 2006; Kluge et al., 2006; Kessler et al., 2011) and regional plot-surveys (Aldasoro et al., 2004), water is the leading determinant for fern richness. Indeed, all of the best performing models in our analyses show that the two richness metrics as well as phylogenetic diversity are explained by annual precipitation combined with a different variable (Table 2). At a global scale, water availability combined with elevation and temperature are most important for richness of pteridophytes (ferns and lycophytes), and also for plants in general (Kreft and Jetz, 2007; Kreft et al., 2010).

A global analysis of pteridophytes indicates that greatest richness occurs in wet tropical regions with increasing elevation, referred to as topographic complexity (Kessler, 2010; Kreft et al., 2010). The role of elevation is confirmed in our analyses with topography combined with annual precipitation best explaining fern richness (but we note that topography is not included the best-performing model in corrected richness and phylogenetic diversity; Table 2). Other regional analyses have also demonstrated the importance of elevation (Dzwonko and Kornaś, 1994; Ferrer-Castan and Vetaas, 2005). It is thought that topography contributes to greater richness because increasing elevations are associated with variability in substrates (including microhabitats), climate, and environment, all yielding a greater diversity of niches for more taxa (Aldasoro et al., 2004; Moran, 2008; Moreno Saiz and Lobo, 2008; Kessler, 2010; Kessler et al., 2011). However, topography alone does not necessarily predict hotspots. In New Zealand, richness is greatest in the North Island (Lehmann et al., 2002), which is considerably less mountainous than the South Island. Instead, the North Island hotspot is associated with the warmest climate zone. At particularly high elevations, the elevational-richness relationship is not observed likely because frost limits fern diversity (Kessler, 2000, 2001; Lehmann et al., 2002; Bhattarai et al., 2004; Grytnes and Beaman, 2006; Kluge et al., 2006; Kessler et al., 2011).

Regions with greater Margalef richness are explained by increases in both precipitation and seasonal temperature extremes. This is in contrast to uncorrected richness, which correlates to increases in precipitation and topography. The difference suggests that sampling biases affect the richness results. These biases may be due to greater sampling effort in topographically complex areas, and the removal of the poorly sampled cells (via the Margalef calculation). The relationship between seasonal temperatures in fern diversity is surprising compared to other findings for ferns (Kessler, 2010) and to trees (Currie and Paquin, 1987). However, seasonality has been identified as a mechanism that enables plants requiring different niches to co-occur (Pausas et al., 2003).

The positive relationship between radiation and phylogenetic diversity suggests that available energy is a limiting factor, conforming to the species-energy hypothesis. Several studies have concluded that plant productivity controls richness (Currie, 1991 and references therein), and for ferns, the link between productivity and species richness has been mechanistically attributed to competition and niche availability (Kessler et al., 2014). However, the mechanisms explaining fern phylogenetic diversity and radiation are unresolved. It has been observed that there is a predominance of epiphytes in tropical rainforests (Gentry and Dodson, 1987; Gentry, 1992). Thus, with low light availability in tropical rainforests, perhaps phylogenetic breadth is limited to epiphytes (which occur in only a few fern clades, Schuettpelz and Pryer, 2009); whereas with greater radiation there is more light available to support a broader range from epiphytic to terrestrial forms. Overall, the relationship between plant phylogenetic diversity and environment is poorly understood with the exception of few analyses (Williams et al., 2010). It remains to be determined if radiation is important for other plant groups, and if radiation is causally linked to phylogenetic diversity.

Interestingly, we found different environmental factors each explaining richness, corrected richness, and phylogenetic diversity. As noted above annual precipitation is common to all of the best models—for richness it combines with topography, for corrected richness it interacts with temperature seasonality, while for phylogenetic diversity it combines with mean annual radiation (Table 2). In general, assessment of fern diversity with environmental factors is conducted for single variables, which unsurprisingly identifies precipitation/humidity as the most important factor (Aldasoro et al., 2004; Bickford and Laffan, 2006; Kessler, 2010). Using two variables, we find that models with single variables were always outperformed by those with two variables (Table 2). We also employed spatial autoregression, which accounted for non-independence of the data by incorporating a spatial term into the models (Dormann et al., 2007; Kissling and Carl, 2007). The models for Margalef richness and phylogenetic diversity were identical using a general linear model and the spatial autoregression model; however, the models differed for richness (Table 2). This result indicates that multivariate models as well as spatial terms need to be considered when inferring the relationship with diversity and environment.

Many fern species distributions are shaped by rock and soil types (Kessler, 2010 and references therein), however, our analyses indicate that fern diversity hotspots are not related to rock and soil substrate (Table 2). In fact, rock grain size, sand, and clay all performed extremely poorly in our models. On the other hand, soil fertility (measured as C/N ratio) best explained fern richness for plots across Uganda (Lwanga et al., 1998). It is possible that fertility may have a strong relationship to richness at the continental-scale, but soil nutrient profiles have been difficult to obtain for larger areas, and future studies are needed to further examine the relationship (Kessler, 2010).

### Differences in phylogenetic representation over the landscape

As discussed above, richness is not necessarily predictive of phylogenetic diversity, despite their general correlation. In this study, we used a randomized phylogenetic diversity test to identify quantitatively places where the expected correlation does not hold. This method discerns whether it has a significantly higher or lower representation of the phylogeny compared to a random sampling of the same number of taxa. In the case of significantly high PD_rand_ (randomized phylogenetic diversity) there is *more* of the phylogeny represented in that grid cell than expected; this is otherwise known as “phylogenetic overdispersion.” Possible explanations include ecological competition that excludes close relatives, or biogeographic processes creating refugia (Webb et al., 2002; Hennequin et al., 2014). Alternatively, when there is significantly low PD_rand_ there is *less* of the phylogeny represented than expected, and this is referred to as “phylogenetic clustering.” Possible explanations include ecological filtering where close relatives have the same habitat requirements, or evolutionarily recent radiations (Webb et al., 2002; Hennequin et al., 2014).

It is also important to note that PD is not necessarily predictive of significant PD_rand_. For example, some regions, such as the Wet Tropics, are high in PD yet significantly low in PD_rand_ (perhaps due to ecological factors as discussed below). Conversely, some areas, such as the Kakadu–Alligator Rivers region in the Northern Territory, have only low to moderate levels of PD, but significantly high PD_rand_ (perhaps due to ecological and biogeographical factors as discussed below). The ability to detect such regions with unusually low or high values of PD shows the value of the PD_rand_ test used here.

We found that there are two strong geographic patterns in the distribution of high and low PD_rand_ cells. The high PD_rand_ cells are clustered in the north of the continent (Figure 2D, purples), whereas the low PD_rand_ cells are concentrated along the east coast (Figure 2D, teals). Given the strong geographic pattern, it is not surprising that there are differences in the environmental conditions between these two regions (Table 3). High PD_rand_ regions are negatively associated with seasonality in temperature, which means that high PD_rand_ is linked to temperature stability. Equable, stable temperatures may be more favorable to a wider range of lineages across the phylogeny because they do not need to evolve adaptations to extremes in temperature.

Significantly low PD_rand_ is positively associated with annual precipitation and temperature seasonality. The latter is indicative of extremes in temperature, however, not all fern clades are able to tolerate cold conditions. Indeed in low PD_rand_ grid cells families that prefer warmer temperatures are absent, such as the Polypodiaceae (with the exception of *Grammatis*, which occur in less than one-quarter of low PD_rand_ cells); while families that are cold tolerant, such as the Blechnaceae and Dennstaedtiaceae are present. Alternatively, the absence of Polypodiaceae may be due to the absence of suitable host trees for this largely epiphytic group. It is also possible that such extremes in conditions limit dispersal due to the absence of suitable traits (Zanne et al., 2014), which for ferns include easily dispersed spores and underground rhizomes, and also promote extinction, although extinction may not necessarily be related to temperature.

## Concluding remarks

Using the largest fern dataset to date, we have identified several areas as hotspots for richness, phylogenetic diversity, and significantly high or low randomized phylogenetic diversity across Australia. Notably, the use of several metrics identifies different or additional areas of importance. The PD_rand_ measure identifies novel areas of diversity significance compared to the two other metrics we used, and these regions have not been revealed in any other analyses. We suggest that these areas are of particular evolutionary and conservation importance and a detailed analysis of them is needed in future studies. Notably environmental predictors explain the distribution of various hotspots, and in turn, the different metrics are predicted by different environmental variables. However, with the onset of changing climate, the conditions that support greatest diversity today will change in the future, e.g., increased and decreased rainfall across Australia (Bureau of Meteorology and CSIRO, 2014), and will likely impact species distributions and thus, diversity patterns.

We note that these broad scale continental-level patterns were obtained using digitized herbarium records. With increasing digitization efforts across the globe we will be able to conduct even larger scale analyses (without having to rely on modeled distributions, Lehmann et al., 2002; Kreft and Jetz, 2007) as has been performed for global marine distribution records (Tittensor et al., 2010). Furthermore, our study is a proof-of-concept that it is possible and vital to incorporate evolutionary metrics when inferring biodiversity hotspots from large compilations of data.

## Author contributions

NSN and BDM designed the study. NSN, NK, and AHT assembled the data. NSN, NK, SWL, and CEG-O analyzed the data. NSN prepared the manuscript, and all authors edited the manuscript.

### Conflict of interest statement

The authors declare that the research was conducted in the absence of any commercial or financial relationships that could be construed as a potential conflict of interest.
