# Supplementary material for: Continental scale patterns and predictors of fern richness and phylogenetic diversity
Source: Front Genet. 2015 Apr 14;6:132. doi: 10.3389/fgene.2015.00132 (PMC4396410; doi:10.3389/fgene.2015.00132)
Supplement: Supplementary file 1 [file DataSheet1.ZIP › Supplemental Data/Figures.pdf]

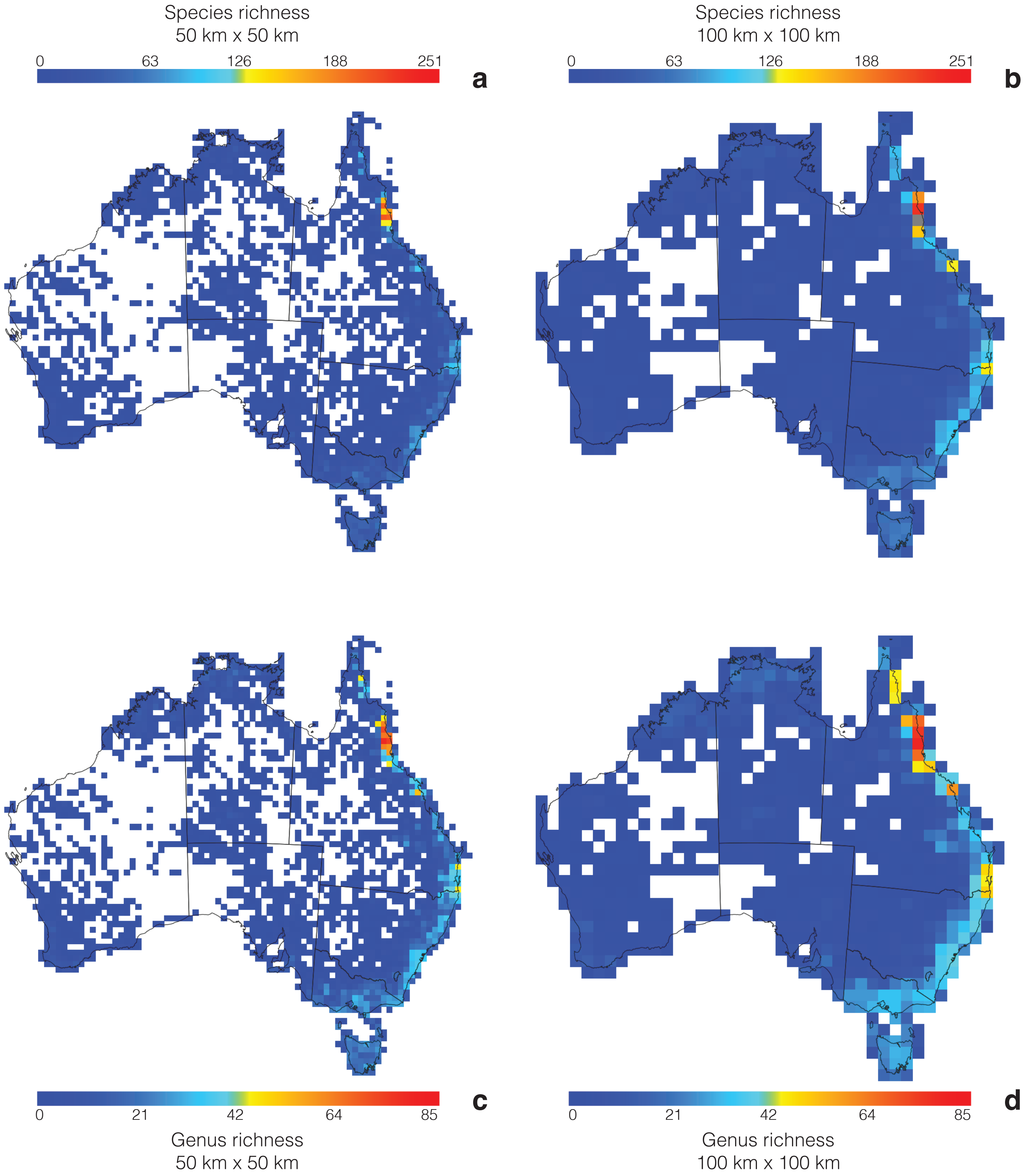


Figure S1 — Nagalingum et al.

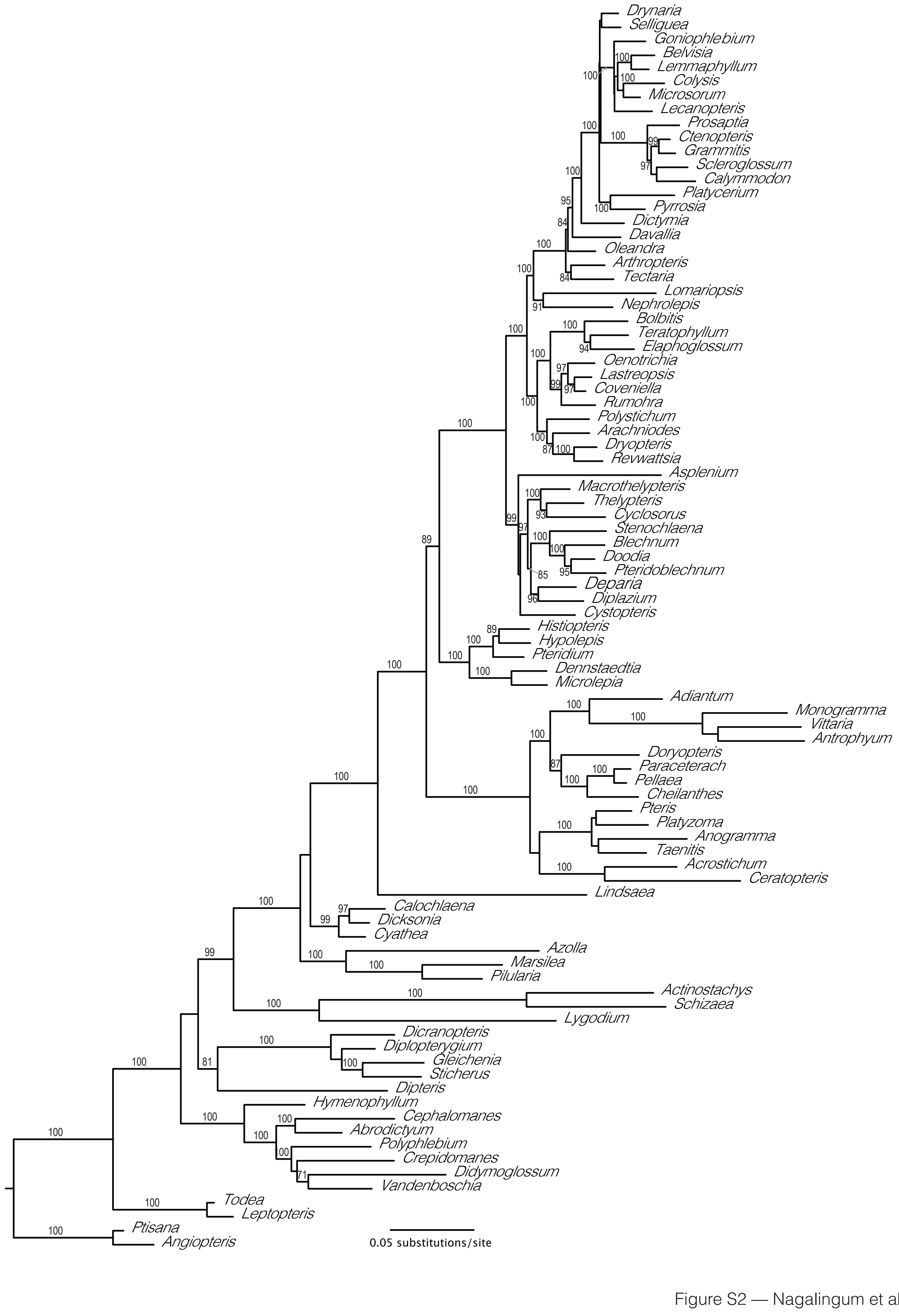


Figure S2 — Nagalingum et al.

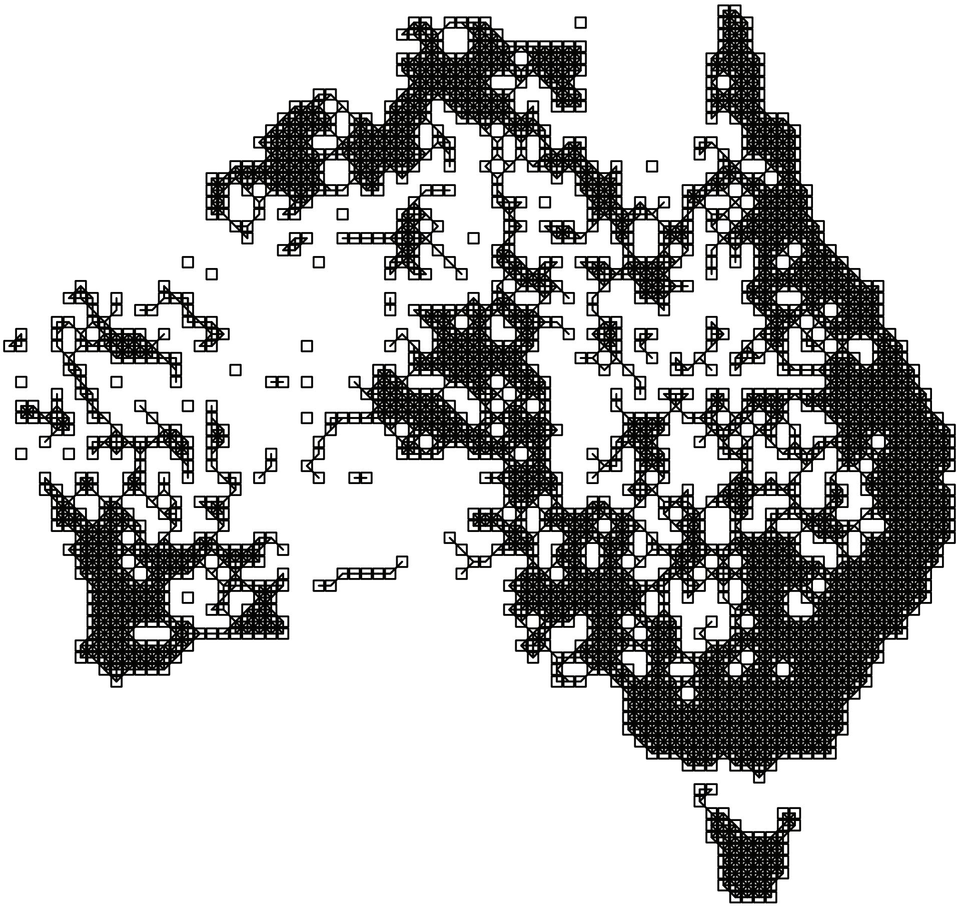

Figure S3 — Nagalingum et al.

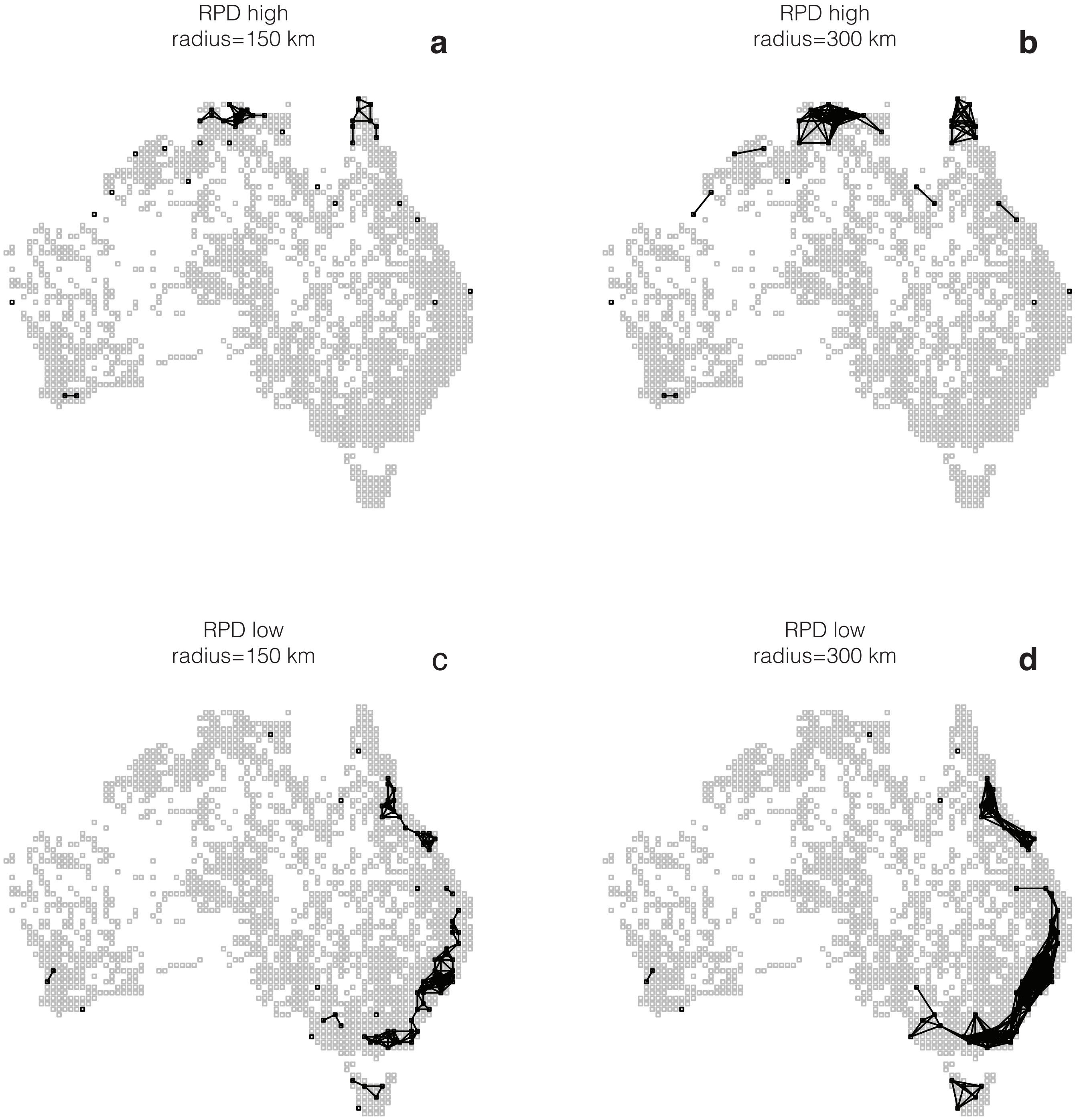


Figure S4 — Nagalingum et al.
