# Supplementary material for: Continental scale patterns and predictors of fern richness and phylogenetic diversity
Source: Front Genet. 2015 Apr 14;6:132. doi: 10.3389/fgene.2015.00132 (PMC4396410; doi:10.3389/fgene.2015.00132)
Supplement: Supplementary file 1 [file DataSheet1.ZIP › Supplemental Data/Figure_Captions.pdf]

SUPPORTING INFORMATION

Figure S1 Comparison of richness patterns using different sized grid cells for species and genera. Species richness using (a) 50 km x 50 km and (b) 100 km x 100 km cells, and genus richness using (c) 50 km x 50 km and (d) 100 km x 100 km cells. The scales for the 50 km x 50 km maps are set according to the upper value of the 100 km x 100 km maps.

Figure S2 Maximum likelihood phylogeny of all fern genera of Australia constructed from a matrix of three molecular markers. Values on branches indicate maximum likelihood bootstrap (MLBS).

Figure S3 Nearest neighbors for the whole dataset showing neighour connections between grid cells. Nearest neighbors were defined by a distance criterion of 75 km—since the grid cells were 50 km x 50 km, all of the eight adjoining grids are in the radius of 75 km. Some grid cells have no nearest neighbours, and are indicated by a lack of connection.

Figure S4 Nearest neighbors for the RPD randomizations of cells that were identified as having high or lower than expected RPD. Cells in black have statistically high or low RPD, and those grey were not statistically significant. The neighbours were defined using two different radius sizes: (a) high RPD using 150 km radius, (b) high RPD using 300 km radius, (c) low RPD using 150 km radius, and (d) low RPD using 300 km radius.
