# Supplementary material for: Continental scale patterns and predictors of fern richness and phylogenetic diversity
Source: Front Genet. 2015 Apr 14;6:132. doi: 10.3389/fgene.2015.00132 (PMC4396410; doi:10.3389/fgene.2015.00132)
Supplement: Supplementary file 1 [file DataSheet1.ZIP › Supplemental Data/Table.pdf]

Table S1 Genera, species and Genbank numbers used to generate the phylogeny used to calculate phylogenetic diversity. "—" indicates no sequence available. Newly sequenced markers for this study are KP164480-KP164497.

Genus	Species and Genbank # for *atpA*	Species and Genbank # for *atpB*	Species and Genbank # for *rbcL*
Abrodictyum	*Abrodictyum elongatum EF463740*	*Abrodictyum elongatum EF463449*	*Abrodictyum brassii AB257483*
Acrostichum	*Acrostichum danaeifolium EF452065*	*Acrostichum danaeifolium EF452008*	*Acrostichum danaeifolium EF473701*
Actinostachys	—	—	*Actinostachys pennula AJ303401*
Adiantum	*Adiantum raddianum EF452071*	*Adiantum raddianum JF935404*	*Adiantum raddianum EF473680*
Angiopteris	*Angiopteris evecta DQ390544*	*Angiopteris evecta EF463485*	*Angiopteris evecta EF463239*
Anogramma	—	*Anogramma leptophylla GU136771*	*Anogramma leptophylla AY168719*
Antrophyum	*Antrophyum latifolium EF452076*	*Antrophyum latifolium EF452018*	*Antrophyum latifolium EF452138*
Arachniodes	*Arachniodes aristata EF463663*	*Arachniodes aristata EF463379*	*Arachniodes aristata AB575069*
Arthropteris	*Arthropteris parallela EF463862*	*Arthropteris parallela EF463522*	*Arthropteris parallela EF463266*
Asplenium	*Asplenium trichomanes EF463613*	*Asplenium trichomanes EF463349*	*Asplenium trichomanes AF525276*
Azolla	*Azolla pinnata EF463859*	*Azolla rubra EF520880*	*Azolla rubra EF520930*
Belvisia	*Belvisia spicata EF463800*	*Belvisia spicata EF463490*	*Belvisia spicata EF463244*
Blechnum	*Blechnum gracile EF463615*	*Blechnum occidentale EU352268*	*Blechnum occidentale AB040565*
Bolbitis	*Bolbitis auriculata EF463665*	*Bolbitis auriculata EF463381*	*Bolbitis auriculata EF463170*
Calochlaena	*Calochlaena villosa EF463658*	*Calochlaena villosa AM176588*	*Calochlaena villosa CVU05912*
Calymmodon	*Calymmodon gracilis EF463801*	*Calymmodon gracilis AY459451*	*Calymmodon gracilis AY362341*
Cephalomanes	*Cephalomanes javanicum EF463741*	*Cephalomanes javanicum EF463450*	*Cephalomanes javanicum Y09195*
Ceratopteris	*Ceratopteris richardii DQ390550*	*Ceratopteris richardii EU352269*	*Ceratopteris thalictroides CTU05609*
Cheilanthes	*Cheilanthes argentea EF452074*	*Cheilanthes distans GU136772*	*Cheilanthes distans GU136791*
Colysis	*Colysis wrightii JF304021*	—	*Colysis wrightii EU482954*
Coveniella	*Coveniella poecilophlebia KP164480*	*Coveniella poecilophlebia KP164487*	*Coveniella poecilophlebia KP164494*
Crepidomanes	*Crepidomanes bipunctatum EF463742*	*Crepidomanes bipunctatum EF463451*	*Crepidomanes bipunctatum EF463227*
Ctenopteris	*Ctenopteris lasiostipes EF463807*	*Ctenopteris heterophylla AY459462*	*Ctenopteris heterophylla AY460629*
Cyathea	*Cyathea speciosa AM176441*	*Cyathea speciosa AM176601*	*Cyathea speciosa AM177339*
Cyclosorus	*Cyclosorus gymnopteridifrons JF304003*	—	*Cyclosorus gymnopteridifrons JF303973*
Cystopteris	*Cystopteris reevesiana EF452088*	*Cystopteris reevesiana EF452028*	*Cystopteris reevesiana EF452149*
Davallia	*Davallia solida EF452089*	*Davallia solida EF452029*	*Davallia solida AY096193*
Dennstaedtia	*Dennstaedtia punctilobula DQ390556*	*Dennstaedtia punctilobula DPU93836*	*Dennstaedtia punctiloba DPU05918*
Deparia	*Deparia lancea EF463908*	*Deparia petersenii EF463568*	*Deparia petersenii AB095977*

Dicksonia	*Dicksonia antarctica* EF463659	*Dicksonia antarctica* DAU93829	*Dicksonia antarctica* EU352301
Dicranopteris	*Dicranopteris linearis* EF463733	*Dicranopteris linearis* GU950634	*Dicranopteris linearis* AB574727
Dictymia	*Dictymia mckeei* EF463810	*Dictymia mckeei* EF463492	*Dictymia brownii* DQ227292
Didymoglossum	*Didymoglossum membranaceum* EF463747	*Didymoglossum membranaceum* EF463456	*Didymoglossum bimarginatum* AB574713
Diplazium	*Diplazium dilatatum* EF463914	*Diplazium dilatatum* EF463573	*Diplazium dilatatum* AB021719
Diplopterygium	*Diplopterygium bancroftii* EF463734	*Diplopterygium bancroftii* AY612695	*Diplopterygium bancroftii* EF463224
Dipteris	*Dipteris conjugata* DQ390559	*Dipteris conjugata* AY612696	*Dipteris conjugata* DCU05620
Doodia	*Doodia media* EF463619	*Doodia media* EF463355	*Doodia media* DMU05922
Doryopteris	*Doryopteris ludens* EF452092	*Doryopteris ludens* EF452031	*Doryopteris ludens* EF452150
Drynaria	*Drynaria rigidula* EF463811	*Drynaria rigidula* EF463493	*Drynaria rigidula* AY529166
Dryopteris	*Dryopteris sparsa* JF304010	*Dryopteris sparsa* EF450497	*Dryopteris sparsa* AB575173
Elaphoglossum	*Elaphoglossum callifolium* JF304013	*Elaphoglossum crassifolium* EF463401	*Elaphoglossum crassifolium* EF463188
Gleichenia	*Gleichenia dicarpa* EF463736	*Gleichenia laevissima* GU950639	*Gleichenia dicarpa* DQ910499
Goniophlebium	*Goniophlebium formosanum* EF463813	*Goniophlebium formosanum* EF463495	*Goniophlebium percussum* AY362561
Grammitis	*Grammitis poeppigiana* EF463819	*Grammitis poeppigiana* AY459478	*Grammitis poeppigiana* AY460646
Histiopteris	*Histiopteris incisa* EF463653	*Histiopteris incisa* EF463374	*Histiopteris incisa* HIU05926
Hymenophyllum	*Hymenophyllum baileyanum* EF463750	*Hymenophyllum baileyanum* EF463459	*Hymenophyllum baileyanum* AF275643
Hypolepis	*Hypolepis tenuifolia* EF463654	*Hypolepis hostilis* HHU93837	*Hypolepis muelleri* HMU05927
Lastreopsis	*Lastreopsis hispida* EF463708	*Lastreopsis hispida* EF463424	*Lastreopsis subrecedens* EF460685
Lecanopteris	*Lecanopteris sinuosa* KP164484	*Lecanopteris sinuosa* KP164491	*Lecanopteris sinuosa* AF470321
Lemmaphyllum	*Lemmaphyllum microphyllum* EF463824	*Lemmaphyllum microphyllum* EF463496	*Lemmaphyllum microphyllum* GQ256314
Leptopteris	*Leptopteris hymenophylloides* EF588675	*Leptopteris superba* DQ646096	*Leptopteris superba* DQ646004
Lindsaea	*Lindsaea blotiana* EF463769	*Lindsaea blotiana* EF463476	*Lindsaea ensifolia* AB574760
Lomariopsis	*Lomariopsis pollicina* EF463776	*Lomariopsis pollicina* EF463481	*Lomariopsis pollicina* HM748135
Lygodium	*Lygodium japonicum* AM176473	*Lygodium japonicum* AM176625	*Lygodium japonicum* AM177360
Macrothelypteris	*Macrothelypteris torresiana* EF463873	*Macrothelypteris torresiana* EF463533	*Macrothelypteris torresiana* EF463277
Marsilea	*Marsilea drummondii* EF463786	*Marsilea drummondii* EU352279	*Marsilea drummondii* DQ643299
Microlepia	*Microlepia speluncae* EF463656	*Microlepia speluncae* EF463377	*Microlepia speluncae* AB574787
Microsorum	*Microsorum grossum* EF463831	*Microsorum grossum* EF463500	*Microsorum grossum* EF463253
Monogramma	*Monogramma graminea* EF452102	*Monogramma graminea* EF452040	*Monogramma acrocarpa* EU024561
Nephrolepis	*Nephrolepis cordifolia* EF452103	*Nephrolepis cordifolia* EF452041	*Nephrolepis cordifolia* HM748146
Oenotrichia	*Oenotrichia tripinnata* KP164482	*Oenotrichia tripinnata* KP164489	*Oenotrichia tripinnata* KP164496
Oleandra	*Oleandra articulata* EF463792	*Oleandra articulata* EF463487	*Oleandra articulata* EF463242
Paraceterach	*Paraceterach muelleri* KP164485	*Paraceterach muelleri* KP164492	*Paraceterach muelleri* KP164497

Pellaea	*Pellaea intermedia* EU268765	*Pellaea falcata* GU136775	*Pellaea falcata* GU136794
Pilularia	*Pilularia globulifera* AM176465	*Pilularia novae zelandiae* EU269700	*Pilularia novae hollandiae* EU269707
Platycerium	*Platycerium stemaria* EF463837	*Platycerium stemaria* EF463506	*Platycerium superbum* DQ164459
Platyzoma	*Platyzoma microphyllum* EF452113	*Platyzoma microphyllum* EF452051	*Platyzoma microphyllum* AY168721
Polyphlebium	*Polyphlebium borbonicum* EF463762	*Polyphlebium endlicherianum* EF463471	*Polyphlebium diaphanum* Y09191
Polystichum	*Polystichum hillebrandii* EF463720	*Polystichum hillebrandii* EF463436	*Polystichum proliferum* AF208393
Prosaptia	*Prosaptia contigua* EF463842	*Prosaptia contigua* AY459494	*Prosaptia contigua* AY362345
Psitana	—	*Marattia oreades* EU439066	*Marattia salicina* EU439085
Pteridium	*Pteridium esculentum* DQ390574	*Pteridium esculentum* PEU93834	*Pteridium esculentum* PEU05940
Pteridoblechnum	*Pteridoblechnum neglectum* KP164483	*Pteridoblechnum neglectum* KP164490	*Pteridoblechnum neglectum* AB040581
Pteris	*Pteris vittata* EF452123	*Pteris vittata* EF452060	*Pteris vittata* AB574846
Pyrrosia	*Pyrrosia polydactyla* EF463844	*Pyrrosia polydactyla* EF463511	*Pyrrosia rupestris* AY362558
Revwattsia	*Revwattsia fragilis* KP164481	*Revwattsia fragilis* KP164488	*Revwattsia fragilis* KP164495
Rumohra	*Rumohra adiantiformis* EF463727	*Rumohra adiantiformis* EF463443	*Rumohra adiantiformis* RAU05648
Schizaea	*Schizaea dichotoma* EF463861	*Schizaea dichotoma* AY612709	*Schizaea dichotoma* AJ303408
Scleroglossum	*Scleroglossum sulcatum* EF463846	*Scleroglossum sulcatum* AY459498	*Scleroglossum sulcatum* AY460664
Selliguea	*Selliguea lanceolata* EF463847	*Selliguea lanceolata* EF463513	*Selliguea lanceolata* EF463261
Stenochlaena	*Stenochlaena tenuifolia* EF463622	*Stenochlaena tenuifolia* EF463358	*Stenochlaena palustris* AB040595
Sticherus	*Sticherus palmatus* DQ390577	*Sticherus palmatus* AY612711	*Sticherus flabellatus* DQ910510
Taenitis	*Taenitis pinnata* KP164486	*Taenitis pinnata* KP164493	*Taenitis blechnoides* TBU05654
Tectaria	*Tectaria apiifolia* EF463866	*Tectaria devexa* EF450520	*Tectaria devexa* D43918
Teratophyllum	*Teratophyllum wilkesianum* EF463730	*Teratophyllum wilkesianum* EF463446	*Teratophyllum wilkesianum* EF463223
Thelypteris	*Thelypteris palustris* EF452127	*Thelypteris palustris* AY612713	*Thelypteris palustris* TPU05947
Todea	*Todea barbara* EF463796	*Todea barbara* AY612714	*Todea barbara* EF588712
Vandenboschia	*Vandenboschia radicans* EF463767	*Vandenboschia radicans* EF463475	*Vandendoschia johnstonense* AB257462
Vittaria	*Vittaria graminifolia* EF452128	*Vittaria flexuosa* JF935442	*Vittaria ensiformis* VEU21290
